# Influence of Microalbuminuria on Long-Term Survival and Cardiovascular or Limb Events in Peripheral Arterial Disease

**DOI:** 10.3400/avd.oa.21-00045

**Published:** 2021-09-25

**Authors:** Kuniki Nakashima, Hisao Kumakura, Ryuichi Funada, Yae Matsuo, Kimimasa Sakata, Akiko Ichikawa, Toshiya Iwasaki, Shuichi Ichikawa

**Affiliations:** 1 Department of Cardiovascular Surgery, Cardiovascular Hospital of Central Japan (Kitakanto Cardiovascular Hospital), Shibukawa, Gunma, Japan; 2 Department of Cardiovascular Medicine, Cardiovascular Hospital of Central Japan (Kitakanto Cardiovascular Hospital), Shibukawa, Gunma, Japan; 3 Department of Nephrology, Cardiovascular Hospital of Central Japan (Kitakanto Cardiovascular Hospital), Shibukawa, Gunma, Japan

**Keywords:** urinary microalbumin, mortality, major adverse cardiovascular events, events of leg

## Abstract

**Objective:** This study aimed to examine the relationship between microalbuminuria and long-term life expectancy or limb events in patients with peripheral arterial disease (PAD).

**Materials and Methods:** A prospective cohort study was performed in 714 patients with PAD. The primary outcomes were cardiovascular or cerebrovascular death (CCVD) and all-cause death (AD), and secondary outcomes were major adverse cardiovascular events (MACE) and cardiovascular and/or limb events (CVLE).

**Results:** The 5, 10, and 15 year survival rates were 82.4%, 53.1%, and 33.0%, respectively. The prevalence of patients with increased microalbuminuria was 39.2%. Higher microalbuminuria, age, C-reactive protein (CRP), lower serum albumin, estimated glomerular filtration rate (eGFR), ankle–brachial pressure index (ABI), diabetes, cerebral infarction, and coronary heart disease (CHD) were associated with CCVD; higher microalbuminuria, age, CRP, D-dimer, lower serum albumin, eGFR, and critical limb ischemia were related to AD; higher microalbuminuria, age, CRP, lower serum albumin, ABI, diabetes, and CHD were related to MACE; higher microalbuminuria, age, lower ABI, cerebral infarction, and CHD were related to CVLE in Cox multivariate analyses (p<0.05). Statins reduced CCVD, AD, MACE, and CVLE (p<0.001).

**Conclusion:** Higher microalbuminuria was a significant predictor for CCVD, AD, MACE, and CVLE in PAD patients.

## Introduction

Peripheral arterial disease (PAD) has been associated with reduced survival because of coronary heart disease (CHD) and cerebral infarction.^1–5)^ PAD is frequently associated with chronic kidney disease (CKD),^6–8)^ which is a risk factor for cardiovascular disease and stroke.^6,9)^ Moreover, increased urinary level of microalbumin (MA) is related to a higher risk of CHD and cardiovascular morbidity.^10)^ In patients with established heart failure, reduced estimated glomerular filtration rate (eGFR) and increased urinary MA are independent predictors for heart failure progression and mortality.^11)^ Hence, CKD is classified on the basis of Cause, eGFR categories, and Albuminuria categories, abbreviated as CGA categories.^12)^

However, long-term cardiovascular or all-cause mortality and cardiovascular or limb event related to the urinary level of MA have not been examined clearly in PAD patients. In the present study, we analyzed the relationship between microalbuminuria and long-term life expectancy or leg event in patients with PAD.

## Materials and Methods

### Subjects

A total of 725 patients with PAD at the Cardiovascular Hospital of Central Japan were recruited from February 1, 2000, and January 31, 2021. Before initiation, the study protocol was approved by the Medical Ethical Committee of our hospital (CCJ-EA-006), and the study was conducted according to the Declaration of Helsinki. The patients received a full explanation of the study and gave written informed consent. The criteria used for PAD included an ankle–brachial pressure index (ABI) of <0.90, clinical symptoms, and iliac or femoropopliteal artery stenosis of ≥70% defined with ultrasound or angiography. Patients with nephrosis syndrome or those on hemodialysis were excluded from this study.

### Baseline patient characteristics

Baseline clinical characteristics for each patient included age, body mass index (BMI), ABI, smoking history, diabetes mellitus (fasting plasma glucose level of >126 mg/dL at least two measurements or a requirement for antidiabetic therapy), and hypertension (receiving oral therapy or blood pressure ≥140/90 mmHg recorded at least twice). A fasting blood sample was collected to measure serum basic metabolic panels. Moreover, we collected a morning urine sample for urinary albumin and creatinine measures. Urinary albumin concentration was measured via turbidimetric immunological technique (Hitachi 7180 automatic analyzer, Hitachi High-Tech Fielding Co., Tokyo, Japan). The urine albumin/creatinine ratio was calculated as mg/g of creatinine (Cr). Patients with an albumin level of <30 mg/gCr were defined as having normoalbuminuria (A1), those with an albumin level of 30–300 mg/gCr (A2), and those with an albumin level of >300 mg/gCr (A3), according to CGA categories.^12)^ All assays were performed at the Health Science Research Institute, Inc. (Saitama, Japan).

An electrocardiogram was recorded for each patient. CHD was determined as a positive sign in stress/rest myocardial perfusion scintigraphy or coronary angiography and a history of this treatment. The eGFR was calculated with the Modification of Diet in Renal Disease equation with Cr level.^13)^

### Endpoints

Each subject was followed up at 1, 2, and 4 months after the procedure and assessed intervals were 4 or 6 months. Medical statuses were evaluated using hospital records and questionnaires for life statuses assessed by the Foot Care Club in our hospital.^3,14,15)^

The definition of myocardial infarction was described previously.^14,16)^ Cerebral infarction was determined as the presence of a new focal neurological deficit confirmed with computed tomography or magnetic resonance imaging. Restenosis was determined as a decrease in ABI of ≥0.15 and ≥50% stenosis on ultrasonography or angiography,^14,17)^ and major amputation was determined as above-the-ankle amputation.

The primary outcomes were cardiovascular or cerebrovascular death (CCVD) and all-cause death (AD), and secondary outcomes were major adverse cardiovascular events (MACE: AD, nonfatal myocardial or cerebral infarction, or transient ischemic attack) and cardiovascular and/or limb events (CVLE: CCVD, nonfatal myocardial or cerebral infarction, transient ischemic attack, major amputation, or any repeat revascularization for a limb).

### Analysis methods

All calculations were performed with IBM SPSS Statistics ver. 25.0 (IBM Corp., Armonk, NY, USA). We used a median (interquartile range) to express continuous variables compared via the Wilcoxon test, and a number (%) to express categorical variables compared via chi-square test. In the follow-up term, the Kaplan–Meier estimates were used to define CCVD, AD, MACE, and CVLE and compared using the log-rank test with Bonferroni correction. In Cox univariate analysis, a hazard ratio and 95% confidence interval were calculated for each factor. In this analysis, risk factors with p<0.05 were used to define significant factors related to outcomes in Cox multivariate analysis. In all analyses, p<0.05 was defined as significant.

## Results

### AD, urinary MA, and characteristics of subjects

Among 725 subjects, follow-up was available for 714 patients. The median follow-up time was 78 (range, 2–238) months. The mean age of the 714 patients was 72.4±9.3 years. The patients who died were 328 (45.9%) throughout the follow-up time. The causes of deaths were cardiovascular disease (n=119, 36.3%), cerebrovascular disease (n=43, 13.1%), malignancy (n=74, 22.6%), pneumonia (n=49, 14.9%), and other causes (n=43, 13.1%). The prevalence of CCVD was 49.4% (n=162). In all patients, the 5, 10, and 15 year freedom from AD rates were 82.4%, 53.1%, and 33.0%, respectively.

Before patients were separated into two subgroups, the prevalence of urinary MA level of A1, A2, or A3 was 60.8% (n=434), 28.2% (n=201), or 11.1% (n=79), respectively. **Table 1** summarizes the baseline clinical characteristics of subjects with normal or increased urinary MA. Patients in A2 or A3 were older; had higher BMI, C-reactive protein (CRP), triglyceride, and D-dimer; and had a lower eGFR. The rates of critical limb ischemia (CLI), stroke, hypertension, and diabetes mellitus were higher in A2 or A3.

**Table 1:** Table 1 Characteristics of subjects in A1 and A2 or A3 categories

	All patients n=714	A1 n=434 (60.8%)	A2 or A3 n=280 (39.2%)	p-value
Age (years)	73 (67–79)	73 (66–79)	74 (68–79)	0.035
Gender (male)	550 (77.0%)	333 (76.7%)	217 (77.5%)	0.442
Body mass index (kg/m^2^)	22.4 (20.2–24.5)	22.1 (19.6–24.1)	22.8 (20.9–25.1)	0.001
Ankle–brachial pressure index	0.68 (0.52–0.82)	0.68 (0.54–0.81)	0.68 (0.49–0.82)	0.432
Critical limb ischemia	107 (15.0%)	51 (11.8%)	56 (20.0%)	0.021
Coronary heart disease	250 (35.0%)	144 (33.2%)	106 (37.9%)	0.115
Cerebral infarction	120 (16.8%)	64 (14.7%)	56 (20.0%)	0.042
Diabetes mellitus	271 (38.0%)	127 (29.3%)	144 (51.4%)	<0.001
Hypertension	483 (67.6%)	275 (63.4%)	208 (74.3%)	0.001
Smoking	540 (75.6%)	330 (76.0%)	210 (75.0%)	0.409
Basic metabolic panel				
Total cholesterol (mg/dL)	191 (164–220)	192 (165–223)	188 (162–217)	0.817
Triglyceride (mg/dL)	130 (94–187)	127 (87–183)	133 (102–189)	0.020
HDL-C (mg/dL)	49 (40–59)	49 (41–59)	47 (39–56)	0.092
LDL-C (mg/dL)	116 (94–137)	117 (95–138)	113 (91–136)	0.631
C-reactive protein (mg/dL)	0.18 (0.08–0.45)	0.16 (0.07–0.43)	0.20 (0.09–0.46)	0.005
D-dimer (µg/dL)	0.8 (0.5–1.7)	0.7 (0.5–1.5)	0.9 (0.5–2.1)	0.016
eGFR (mL/min/1.73 m^2^)	60.4 (49.1–72.4)	63.4 (54.5–73.7)	54.4 (41.9–71.1)	<0.001
Urinary albumin (mg/gCr)	18.0 (6.8–83.5)	8.3 (4.9–15.3)	108.1 (52.3–340.9)	<0.001
Serum albumin (g/dL)	4.0 (3.8–4.2)	4.0 (3.8–4.3)	4.0 (3.8–4.2)	0.057
Revascularization	458 (64.1%)	286 (65.9%)	172 (61.4%)	0.128
Medications				
Thienopyridines	264 (37.0%)	166 (38.2%)	98 (35.0%)	0.212
Aspirin	424 (59.4%)	263 (60.6%)	161 (57.5%)	0.228
Beraprost	287 (40.2%)	171 (39.4%)	116 (41.4%)	0.322
Cilostazol	165 (23.1%)	100 (23.0%)	65 (23.2%)	0.514
Ca antagonist	359 (50.3%)	206 (47.5%)	153 (54.6%)	0.061
β-blocker	100 (14.0%)	55 (12.7%)	45 (16.1%)	0.122
ARB	231 (32.4%)	137 (31.6%)	94 (33.6%)	0.316
ACE inhibitor	76 (10.6%)	40 (9.2%)	36 (12.9%)	0.079
Statin	464 (65.0%)	288 (66.4%)	176 (62.9%)	0.338

A1: urinary albumin levels less than 30 mg/gCr (normoalbuminuria), A2: urinary albumin levels with 30–300 mg/gCr, A3: urinary albumin levels more than 300 mg/gCr HDL-C: high-density lipoprotein cholesterol; LDL-C: low-density lipoprotein cholesterol; eGFR: estimated glomerular filtration rate; ARB: angiotensin receptor blocker; ACE: angiotensin-converting enzyme

### Factors for CCVD and AD

**Figure 1:**
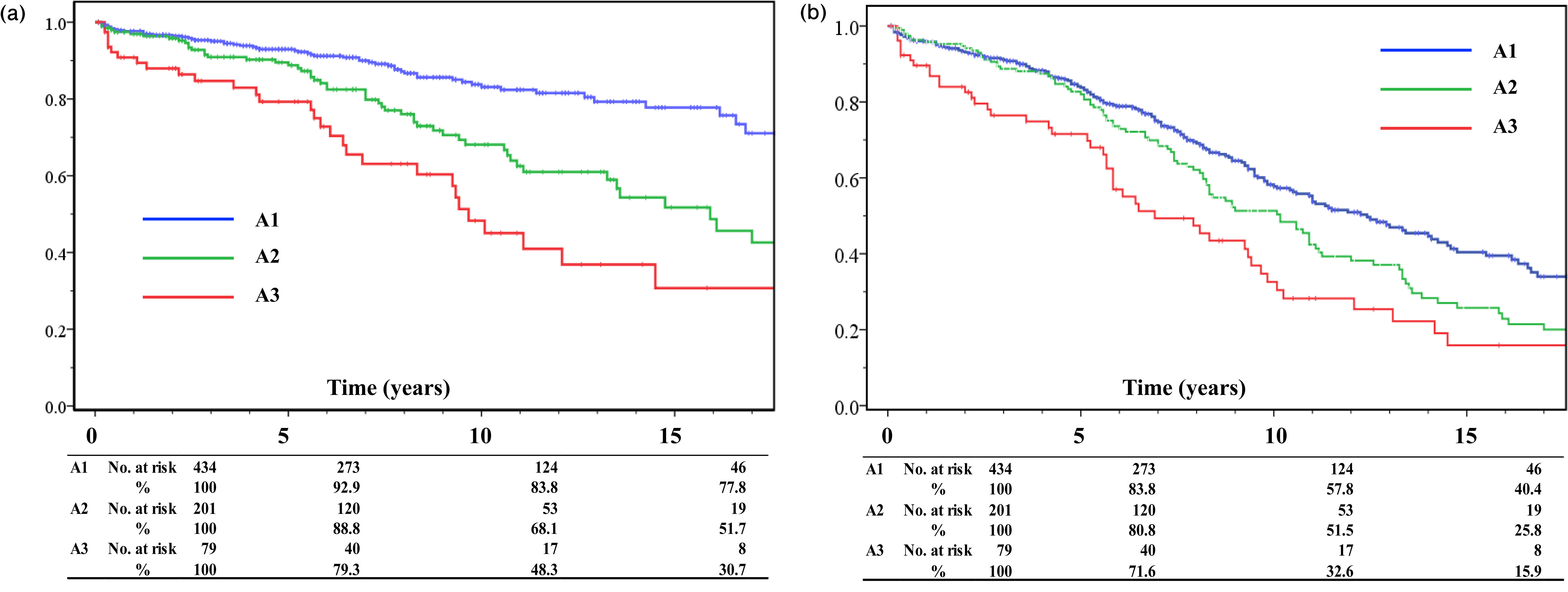
Fig. 1 **(a)** Freedom from cardiovascular or cerebrovascular death (CCVD) in A1, A2, or A3 category. There were significant differences among A1, A2, or A3 categories (p-values were <0.001, <0.001, and 0.009 in A1 vs. A2, A1 vs. A3, and A2 vs. A3, respectively). **(b)** Freedom from all-cause death (AD) in A1, A2, or A3 category. There were significant differences among A1, A2, and A3 categories (p-values were 0.048, <0.001, and 0.039 in A1 vs. A2, A1 vs. A3, and A2 vs. A3, respectively).

The 5, 10, and 15 year rates for freedom from CCVD among A1, A2, or A3 with significant differences (p<0.01) are shown in **Fig. 1(a)**. Higher level of urinary MA, age, CRP, D-dimer, lower level of serum albumin, BMI, ABI, eGFR, CLI, diabetes, cerebral infarction, and CHD were related to CCVD in Cox univariate analysis (**Table 2(a)**, p<0.05). Statin was related to CCVD. Higher urinary level of MA, age, CRP, lower level of serum albumin, eGFR ABI, diabetes, cerebral infarction, and CHD were also associated with CCVD, and statin reduced CCVD in Cox multivariate analysis (p<0.05).

**Table 2a:** Table 2(a) Univariate and multivariate analyses for cardiovascular or cerebrovascular death

	Univariate			Multivariate		
	HR	95%CI	p-value	HR	95%CI	p-value
Age (years)	1.066	1.046–1.087	<0.001	1.045	1.023–1.068	<0.001
Sex (male)	1.049	0.693–1.586	0.822			
Body mass index (kg/m^2^)	0.902	0.855–0.953	<0.001	0.965	0.907–1.027	0.265
Ankle–brachial pressure index	0.265	0.146–0.478	<0.001	0.431	0.205–0.902	0.026
Critical limb ischemia	3.004	2.010–4.490	<0.001	1.279	0.956–1.711	0.098
Diabetes mellitus	1.699	1.222–2.362	0.002	1.570	1.048–2.350	0.029
Cerebral infarction	2.371	1.629–3.451	<0.001	1.602	1.025–2.504	0.039
Coronary heart disease	1.543	1.136–2.178	0.006	1.482	1.001–2.194	0.048
Urinary albumin (mg/gCr)	1.001	1.001–1.001	<0.001	1.001	1.000–1.001	0.020
Serum albumin (g/dL)	0.225	0.153–0.330	<0.001	0.432	0.266–0.703	0.001
eGFR (mL/min/1.73 m^2^)	0.975	0.966–0.987	<0.001	0.989	0.979–1.000	0.042
C-reactive protein (mg/dL)	1.178	1.084–1.280	<0.001	1.168	1.046–1.305	0.006
D-dimer (µg/dL)	1.017	1.002–1.032	<0.001	1.006	0.983–1.030	0.615
Statin	0.308	0.213–0.445	<0.001	0.450	0.296–0.686	<0.001

HR: hazard ratio; CI: confidence interval; eGFR: estimated glomerular filtration rate

The 5, 10, and 15 year rates for freedom from AD among A1, A2, and A3 with significant differences (p<0.05) are shown in **Fig. 1(b)**. Higher level of urinary MA, age, CRP, D-dimer, lower level of serum albumin, BMI, ABI, eGFR, and CLI were related to AD in Cox univariate analysis (**Table 2(b)**, p<0.05). Revascularization, statin, or aspirin was related to AD. Higher urinary level of MA, age, CRP, D-dimer, lower level of serum albumin, eGFR, and CLI were also associated with AD, and statin reduced AD in multivariate analysis (p<0.05).

**Table 2b:** Table 2(b) Univariate and multivariate analyses for all-cause death

	Univariate			Multivariate		
	HR	95%CI	p-value	HR	95%CI	p-value
Age (year)	1.076	1.062–1.090	<0.001	1.054	1.039–1.070	<0.001
Sex (male)	1.226	0.943–1.701	0.117			
Body mass index (kg/m^2^)	0.897	0.865–0.930	<0.001	0.964	0.927–1.002	0.061
Ankle–brachial pressure index	0.340	0.226–0.512	<0.001	0.709	0.432–1.162	0.173
Critical limb ischemia	2.533	1.901–3.375	<0.001	1.517	1.049–2.193	0.027
Urinary albumin (mg/gCr)	1.001	1.000–1.001	<0.001	1.001	1.000–1.001	0.045
Serum albumin (g/dL)	0.257	0.196–0.336	<0.001	0.460	0.331–0.639	<0.001
eGFR (mL/min/1.73 m^2^)	0.966	0.980–0.992	<0.001	0.993	0.987–0.999	0.034
C-reactive protein (mg/dL)	1.175	1.113–1.241	<0.001	1.064	1.080–1.255	<0.001
D-dimer (µg/dL)	1.021	1.012–1.029	<0.001	1.011	1.000–1.022	0.047
Aspirin	0.781	0.627–0.953	0.028	0.805	0.813–1.353	0.715
Statin	0.463	0.357–0.570	<0.001	0.462	0.353–0.605	<0.00
Revascularization	0.711	0.564–0.896	0.004	0.805	0.610–1.063	0.126

HR: hazard ratio; CI: confidence interval; eGFR: estimated glomerular filtration rate

### Factors for MACE and CVLE

**Figure 2:**
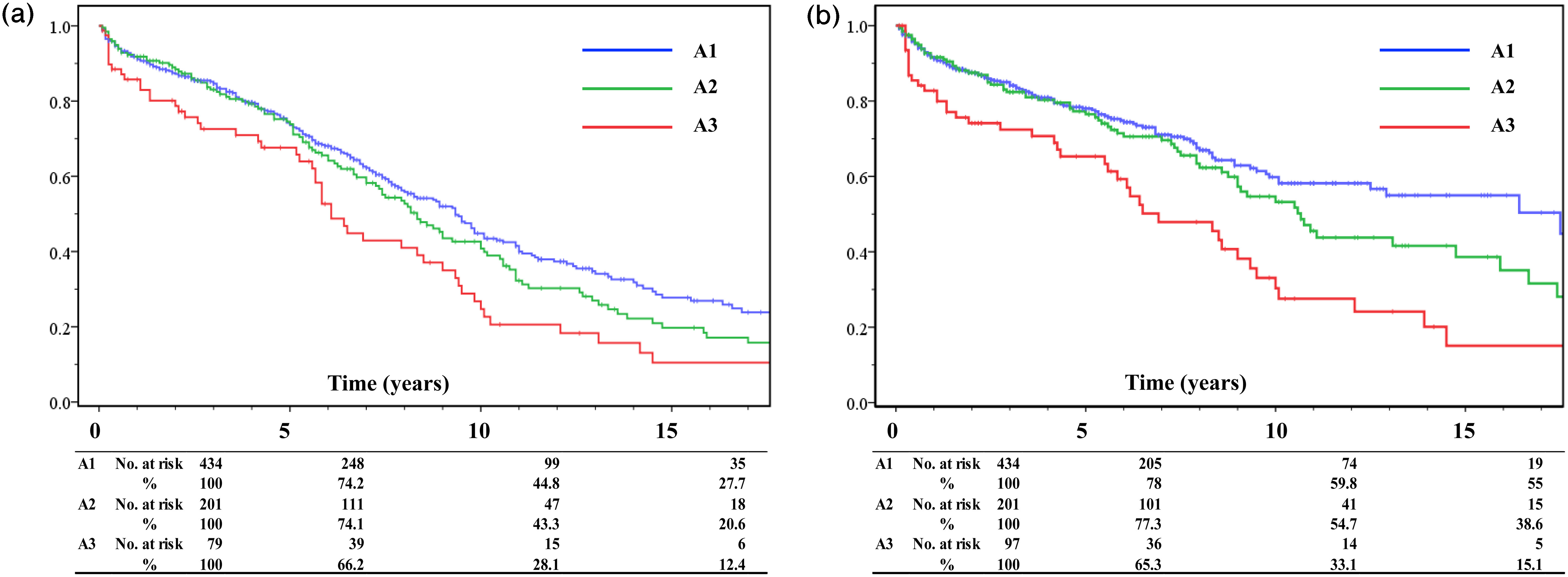
Fig. 2 **(a)** Freedom from major adverse cardiovascular events (MACE) in A1, A2, or A3 category. There were significant differences in A1 versus (vs.) A3 (p=0.009) and A2 vs. A3 categories (p=0.039), but there was no significant difference in A1 vs. A2 categories (p=0.141). **(b)** Freedom from cardiovascular and/or limb events (CVLE: cardiovascular or cerebrovascular death, nonfatal myocardial or cerebral infarction, transient ischemic attack, major amputation, or repeat revascularization for a limb) in A1, A2, or A3 category. There were significant differences in A1 vs. A3 (p<0.001) and A2 vs. A3 categories (p=0.006), but there was no significant difference in A1 vs. A2 categories (p=0.126).

The 5, 10, and 15 year rates for freedom from MACE are shown in **Fig. 2(a)**. Significant differences were found between A1 and A3 (p=0.009) and A2 and A3 categories (p=0.039), but there was no significant difference between A1 and A2 categories (p=0.141). Higher level of urinary MA, age, CRP, D-dimer, lower level of serum albumin, BMI, ABI, eGFR, CLI, diabetes, and CHD were related to MACE, and statin therapy was related to MACE significantly in Cox univariate analysis (**Table 3(a)**, p<0.05). Higher level of urinary MA, age, CRP, lower level of serum albumin, ABI, diabetes, and CHD were related to MACE, and statin therapy was related to MACE in multivariate analysis (p<0.05).

**Table 3a:** Table 3(a) Univariate and multivariate analyses for major adverse cardiovascular events

	Univariate			Multivariate		
	HR	95%CI	p-value	HR	95%CI	p-value
Age (years)	1.050	1.038–1.061	<0.001	1.042	1.030–1.055	<0.001
Body mass index (kg/m^2^)	0.945	0.916–0.976	0.001	0.992	0.958–1.027	0.663
Ankle–brachial pressure index	0.397	0.277–0.568	<0.001	0.610	0.393–0.947	0.028
Critical limb ischemia	2.183	1.681–2.835	<0.001	1.251	0.901–1.737	0.181
Diabetes mellitus	1.348	1.101–1.650	0.004	1.332	1.053–1.666	0.017
Coronary heart disease	1.731	1.421–2.108	<0.001	2.152	1.724–2.686	<0.001
Urinary albumin (mg/gCr)	1.001	1.000–1.001	<0.001	1.000	1.000–1.001	0.033
Serum albumin (g/dL)	0.356	0.281–0.452	<0.001	0.625	0.470–0.832	0.001
eGFR (mL/min/1.73 m^2^)	0.991	0.995–0.996	0.001	1.000	0.995–1.006	0.959
C-reactive protein (mg/dL)	1.133	1.073–1.195	<0.001	1.113	1.037–1.194	0.003
D-dimer (µg/dL)	1.016	1.007–1.025	<0.001	1.010	0.998–1.022	0.089
Statin	0.372	0.302–0.459	<0.001	0.422	0.334–0.535	<0.001

HR: hazard ratio; CI: confidence interval; eGFR: estimated glomerular filtration rate

The 5, 10, and 15 year rates for freedom from CVLE are shown in **Fig. 2(b)**. There were significant differences between A1 and A3 (p<0.001) and A2 and A3 categories (p=0.006), but no significant difference was found between A1 and A2 categories (p=0.126). Higher level of urinary MA, age, CRP, lower level of serum albumin, ABI, eGFR, CLI, diabetes, cerebral infarction, and CHD were related to CVLE, and statin therapy was related to CVLE in Cox univariate analysis (**Table 3(b)**, p<0.05). Higher urinary level of MA, age, lower ABI, cerebral infarction, and CHD were related to CVLE, and statin therapy was also related to CVLE in multivariate analysis (p<0.05). The higher urinary level of MA was a significant factor associated with CVLE in this analysis (p=0.007).

**Table 3b:** Table 3(b) Univariate and multivariate analyses for cardiovascular and/or limb events (CVLE: cardiovascular or cerebrovascular death, nonfatal myocardial or cerebral infarction, transient ischemic attack, major amputation, or repeat revascularization for a limb)

	Univariate			Multivariate		
	HR	95%CI	p-value	HR	95%CI	p-value
Age (years)	1.019	1.005–1.032	0.007	1.016	1.001–1.031	0.039
Ankle–brachial pressure index	0.383	0.245–0.599	<0.001	0.452	0.261–0.781	0.004
Critical limb ischemia	1.857	1.316–2.621	<0.001	1.347	0.980–1.851	0.066
Diabetes mellitus	1.433	1.109–1.853	0.006	1.278	0.962–1.698	0.091
Cerebral infarction	1.632	1.187–2.242	0.003	1.450	1.016–2.069	0.041
Coronary heart disease	2.175	1.687–2.805	<0.001	2.091	1.575–2.776	<0.001
Urinary albumin (mg/gCr)	1.001	1.000–1.001	<0.001	1.001	1.000–1.001	0.007
Serum albumin (g/dL)	0.498	0.365–0.679	<0.001	0.797	0.555–1.143	0.217
eGFR (mL/min/1.73 m^2^)	0.990	0.983–0.997	0.003	1.000	0.993–1.008	0.918
C-reactive protein (mg/dL)	1.087	1.002–1.179	0.045	1.052	0.955–1.159	0.306
Statin	0.479	0.363–0.621	<0.001	0.515	0.386–0.687	<0.001

HR: hazard ratio; CI: confidence interval; eGFR: estimated glomerular filtration rate

## Discussion

Our data represented the first clinical evidence for the relationship between microalbuminuria and long-term life expectancy or leg events in PAD patients. In this study, the prevalence of albuminuria categories (A2 or A3) was 39.2%, and the higher level of urinary MA was a predictive factor for CCVD and AD. In this study, CCVD was correlated with the higher urinary level of MA, age, CRP, lower level of serum albumin, eGFR, ABI, diabetes, cerebral infarction, and CHD. And AD was correlated with the higher urinary level of MA, age, CRP, D-dimer, lower level of serum albumin, eGFR, and CLI. Some studies have reported that CKD is a risk factor for CHD, valvular heart disease, and cerebrovascular disease,^6,9,18)^ and cardiovascular death is markedly accelerated in patients on hemodialysis.^6)^ Moreover, microalbuminuria is independently related to increased cardiovascular risks and morbidity.^10)^ We have reported that urinary level of MA is related to low-density lipoprotein cholesterol, age, CHD, CLI, and diabetes in patients with PAD.^8)^ Hence, CKD is classified on the basis of eGFR categories, and urinary levels of MA are added in CGA categories.^12)^

CLI and lower ABI are associated with a higher risk of cardiovascular events because of systemic or severe atherosclerosis.^3,19)^ Cardiovascular and cerebrovascular diseases were major causes of AD. Moreover, malnutrition is a strong risk factor for AD.^3,20)^ We have reported that the geriatric nutritional risk index is a meaningful predictor for AD, MACE, and major adverse cardiovascular and limb events in PAD patients.^21)^ These results suggest that patients with lower serum albumin, lower ABI, or CLI have causes of higher mortality based on systemic severe atherosclerosis.

In this study, higher albuminuria was also an independent predictive factor for MACE and CVLE. Higher level of urinary MA, age, CRP, lower level of serum albumin, ABI, diabetes, and CHD were related to MACE, and higher urinary MA level, age, lower ABI, cerebral infarction, and CHD were related to CVLE. Microalbuminuria has been reported to be independently related to increased cardiovascular risk factors and MACE.^10)^ Moreover, reduced eGFR and albuminuria are independent predictors of heart failure progression and mortality in patients with heart failure.^11)^ We have reported that cerebral infarction, diabetes, and CKD are predictive risk factors for aggravation of stages of PAD.^15,22)^ Sensory disturbance, decreased physical faculties, and systemic atherosclerosis may be related to the causes of these outcomes.^1,22)^ Urinary MA levels are associated linearly with an increasing likelihood for the presence of PAD, even in the normal range of albuminuria in the Japanese general population.^23)^ Systemic vascular atherosclerosis reflected by a higher level of urinary MA might be responsible for MACE and CVLE. In this study, a higher level of urinary MA was a significant risk factor associated with CVLE. Hence, higher urinary MA level was related to systemic vascular atherosclerosis including limb vessels in patients with PAD.

Statin therapy has an antiatherogenic effect on CHD and reduces MACE.^24,25)^ Treatments with statins are efficient for improving AD and MACE in PAD patients with or without symptoms detected via the measurements of ABI.^26)^ However, treatments with statins appear to be less effective in decreasing risks of CHD in patients with CKD and those on hemodialysis treatment.^27,28)^ Additionally, statins are recommended for all patients with PAD in recent guidelines.^29)^ Our findings also suggested that statins improve long-term CCVD and AD and decrease the risks of MACE and CVLE in patients with PAD.

The present study had some limitations: 1) the number of patients was relatively small; 2) this study was based on the data from a single institution; 3) a single morning urine sample was used to measure albumin and creatinine levels; 4) the prevalence of CLI was low as a result of the inclusion criteria in which patients on hemodialysis were excluded; and 5) the rate of statin therapy was relatively lower than in the recent guidelines, but the rate has risen over time. These limitations need further study with a larger patient cohort with PAD for CCVD, AD, MACE, and CVLE.

## Conclusion

The urinary level of MA was a significant predictor for CCVD, AD, MACE, and CVLE in patients with PAD.
